# Screening of anti-microbial, anti-biofilm activity, and cytotoxicity analysis of a designed polyherbal formulation against shigellosis

**DOI:** 10.1016/j.jaim.2021.06.007

**Published:** 2021-11-09

**Authors:** Devendra Singh, Deepmala Sharma, Vishnu Agarwal

**Affiliations:** a Department of Biotechnology Motilal Nehru National Institute of Technology, Allahabad, 211004, U.P., India; b Department of Mathematics, National Institute of Technology, Raipur, India

**Keywords:** Shigellosis, *Shigella flexneri*, Formulation, Antibiofilm, Anti-quorum sensing

## Abstract

**Background:**

Shigellosis is an infectious intestinal disease common in rural communities. In developing countries, shigellosis is caused predominantly by *Shigella flexneri* and has been determined as a major cause of morbidity and mortality.

**Objective:**

The study investigates the anti-biofilm, anti-microbial, and anti-shigellosis activity of a designed formulation.

**Materials and methods:**

The potential of the formulation against *S. flexneri* (MTCC 1457) was investigated using a well-diffusion assay. Further, the effect of the designed formulation on bacterial growth and biofilm formation was analyzed by the spectrophotometry method. Anti-quorum sensing activity was analyzed using *Chromobacterium violaceum* CV026, a bacterial strain. Finally, the cytotoxicity of the formulation was examined by using cell line and brine shrimp lethality assay.

**Results:**

The MIC value of the aqueous extract of the formulation was 2.4 mg ml^−1^ and an inhibitory zone of 23 mm was observed. On the other side, the formulation significantly inhibited the bacterial growth, biofilm formation (23.78%), violacein inhibition (27.68%) at 0.6 mg ml^−1^ concentration (did not significantly affect the growth curves) and was found non-toxic in cell assay and brine shrimp lethality assay.

**Conclusion:**

According to the result obtained, the designed formulation was found effective and non-toxic, so it can be used to treat shigellosis and *Shigella*-related infections.

## 1 Introduction

Shigellosis (bacillary dysentery) is one of the important enteric infectious diseases caused by the *Shigella* species. It is a non-motile, facultatively anaerobic, rod-shaped, gram-negative bacteria and is associated with bacterial species belonging to the Enterobacteriaceae family [1,2]. The major characteristic clinical symptoms of shigellosis include bloody, watery, mucoid stools in which the passage of frequent small-volume stools is associated with variable degrees of fever, fecal urgency, and systemic toxicity. *Shigella flexneri* tend to be the predominant bacteria in causing bacillary dysentery than other major bacteria, including *Escherichia* *coli* and *Salmonella*, in developing countries [3]. There are four *Shigella* species (*Shigella dysenteriae, Shigella bodyii, S. flexneri,* and *Shigella sonnei*), which are mainly responsible for causing bacillary dysentery. In India (eastern India), *S. flexneri* (60.7%) is the most prevalent one, followed by *S. sonnei* (23.8%), *S.* *dysenteriae* (9.8%), and finally *S.* *boydii* (5.7%) serogroup. Interestingly in South India, *S. flexneri* (> 90%) is found again to be the most prevalent one, followed by *S. sonnei* (3.9–5.4%) [1]. Among those species, *S. flexneri* (74.7%) are majorly responsible for shigellosis, among the children below the age of 5 years, with approximately more than 10 million cases every year [4]. It invades and exploits the intestinal epithelial cells, which will result in the stimulation of severe mucosal inflammation [4]. Thus, *Shigella* infection will ultimately result in rectum and colon tissue destruction in humans [3]. Due to improper care, shigellosis may lead to a life-threatening systemic disease known as a hemolytic uremic syndrome, characterized by hemolytic uremia, thrombocytopenia, and kidney failure. Approximately 2–3% of *S. flexneri* infected individuals may develop eye irritation, pain during urination, and joint pains [5]. This situation is termed Reiter's syndrome, which may lead to a chronic arthritis problem [6].

Different antibiotics (such as a fluoroquinolone, sulfamethoxazole, ampicillin, ciprofloxacin, and nalidixic acid) are prescribed for the treatment of shigellosis. But, the increasing number of antibiotic-resistant bacteria is becoming a global problem [7].

Biofilm formation in bacteria is a complex process that includes EPS secretion, adherence, and detachment from matured biofilm and is principally connected to bacterial quorum sensing (QS). Numerous research studies have shown that *Shigella* forms biofilms under different conditions, protecting it from stress situations and making it more resistant towards the anti-bacterial agents [[8], [9], [10]]. QS is a process of bacterial cell-to-cell communication. This process is utilized by the bacteria to switch on the various gene expression programs related to population density. Thus, designing a drug or formulation having anti-QS activity will be more effective [11].

Due to such concerns, there is a necessity to develop a natural and potentially effective formulation [12]. In fact, plant extracts are highly rich in phytochemical and give a naturally produced, biologically active synergistic composition. Plant extracts and polyherbal formulation has been commonly used to treat various disease from ancient times. Due to the presence of various chemical compounds (like tannin, flavonoids, polyphenols), the extracts of *Citrus lemon* fruit*, Camellia sinensis* leaf, *Phyllanthus emblica* fruit, *Terminalia chebula* fruit, and *Terminalia arjuna* bark were traditionally used to treat various diseases and its related infections. This is the first time an herbal formulation of *C. sinensis, T. chebula, C. lemon, P. emblica,* and *T. arjuna* was designed for the treatment of shigellosis. These synergistic compositions could be more effective in comparison with the single molecules and are least likely to elicit the development of resistance [13].

## 2 Methods

### 2.1 Plant collection

Dried powdered extract of *C. sinensis* leaf (Batch no. CS/0010416), *T. chebula* fruit (Batch no. KP/TC/001/16), *P. emblica* fruit (Batch no. PE/0010516), *T. arjuna* bark (Batch no. NBT/1705577) and *C. lemon* fruit (Batch no. NBT/1705576) were obtained from K. Patel Phyto Extractions Pvt. Ltd (Gujarat, India) and Saamir international Pvt. Ltd (Delhi, India) respectively.

### 2.2 Bacteria and growth condition

The bacterial strain of *S. flexneri* (MTCC 1457) was procured from MTCC and was grown in nutrient broth media at 37 °C, pH 7.0. When the OD reached 1 at 600 nm, the cultures were sub-cultured. Cell line SW480 was procured from National Centre for Cell Science (NCCS), Pune. Media, including NB, TSB, and artificial sea salt, was purchased from Himedia. *Chromobacterium violaceum* CV026 (ATCC31352) was procured from CECT, Spain, and was grown at 30 °C. HHL (hexanoyl homoserine lactone) and Kanamycin were purchased from Sigma–Aldrich, India, supplemented in CV026 plates.

### 2.3 Preparation of formulation

The individual extracts efficacy was analyzed and on the basis of which desired amount of powdered extracts of different plant parts viz. *C. sinensis* 24% (leaf), *P. emblica* 20% (fruit), *C. lemon* 20% (fruit), *T. arjuna* 18% (bark), *T. chebula* 18% (fruit) were taken and mixed by a mechanical stirrer until the mixture was homogeneous. After mixing in the aqueous solvent (100 mg.ml-1 stock solution), the obtained formulation was kept at -4 °C.

### 2.4 MIC (Minimum Inhibitory Concentration)

The MIC of formulations against *S. flexneri* was analyzed by using the broth dilution method as per *NCCLS*, USA, 2006 guidelines [14]. In brief, *S. flexneri* culture (OD 600 nm = 1) was grown at 37 °C for 24 h in the nutrient broth and mixed with varying concentrations of formulation in 96 well plates. The lowest concentration, which completely inhibits bacterial growth, was noted as the MIC for the formulation, and further analysis was carried out at sub-MIC concentration [11].

### 2.5 Growth curve analysis

To determine the effect of formulation (sub-lethal concentrations) on the *S. flexneri* and *C. violaceum* CV026 strains, a growth curve study was performed. Briefly, overnight cultures of *S. flexneri* and *C. violaceum* CV026 strains were inoculated into LB broth medium (100 ml) along with varying concentrations of formulation (control, 0.3, 0.6, 0.9, and 1.2 mg ml^−1^). The flasks were put in the incubator at the optimal temperatures for the respective bacterial strains, and OD at 600 nm was noted at 2 h intervals till 24 h [11,15].

### 2.6 Well diffusion assay

Agar well diffusion is a widely accepted and commonly used method for assessing anti-microbial activity. In brief, on the prepared agar plate surface, the bacterial inoculums were spread by using a sterile spreader. Then, aseptically a well is punched with a sterile cork borer or a tip, and a sample volume (100 μL) of the formulation of the desired concentration is placed into the created well. Further, these agar plates are incubated at 37 °C for 24 h. The clear inhibition halo zone was observed, which shows the formulation's anti-microbial activity against the *S. flexneri* strain. The diameter of the clear halo zone was measured in mm [16].

### 2.7 Quantification of biofilm

Biofilms were allowed to form under the absence or at sub-lethal concentrations of formulations in 96-well microtiter plates. In brief, 100ul of TSB media was added to U-shaped 96 well plates, along with the formulations in all wells except the control well. Further, 1 μl of 0.5 OD overnight bacteria culture was added in each well and left for 24 h incubation at 37 °C. After incubation, non-adherent or planktonic cells were discarded, and the plate was gently washed (3 times) with PBS and dried at 37 °C. After that, each well was stained by adding 200 μl of 0.5% Crystal Violet and left for 15 min. After 15 min, the stain was discarded, and again the wells were washed by using PBS so that the excess stain will be removed. Finally, after washing, DMSO was added to solubilize the stain (Crystal Violet), and the plates were left for 5–8 min, and OD was taken at 590 nm in a microplate reader. The experiment was done in triplicate [11].

% Biofilm inhibition = (mean OD590 control - mean OD 590 sample / Mean OD 590 Control)∗100

### 2.8 Violacein inhibition assay

#### 2.8.1 Spread plate method

Concisely, 100 μl of CV026 culture (OD1 at 600 nm) was spread by using a sterile glass spreader on LB agar prepared plates having an HHL and Kanamycin. After 5 min, wells were cut aseptically by using a tip. Formulation dissolved in an aqueous solvent was placed into the specified wells and left for incubation for 24 h at 28 °C. Finally, the plates were observed for the halo zone formed along with the wells with purple backgrounds [11].

#### 2.8.2 Quantitative inhibition

A 100 μl of *C. violaceum* CV026 overnight culture (OD 1 at 600 nm) having HHL and Kanamycin was added to the 96-well plate wells with 100 μl of LB broth and incubated in the absence or presence of varying concentrations of designed formulation (0.3, 0.6, 0.9, 1.2 mg ml^−1^). Then, the plate was incubated for 24 h at 28 °C and then dried completely at 60 °C. Then, 100 μl DMSO was added to the wells and was incubated at 30 °C with shaking. Finally, the absorbance was taken with the help of an ELISA plate-reader at 585 nm [15].

### 2.9 Cytotoxicity analysis

#### 2.9.1 Cell line

SW480 (human colon cancer) cell line was procured from NCCS Pune. SW480 cells were grown in DMEM (Dulbecco's modified Eagle's medium) along with 10% FBS (Fetal bovine serum, Gibco) and 1% penicillin-streptomycin (pen strep) antibiotic at 37 °C under a humidified atmosphere of 5% CO2. For cytotoxic analysis, a stock solution of the formulation was prepared by dissolving with culture medium and was determined using MTT assay. The human colon cancer cell line, after washing with PBS, was incubated for a few minutes with 0.25% trypsin–EDTA. Now, the harvested cells were added at a density of 1 × 10^4^ cells per well on 96-well plates. After the completion of 24 h, cells were treated with varying concentrations of formulations, including 10 μg ml^−1^, 25 μg ml^−1^, 50 μg ml^−1^, 100 μg ml^−1^, and incubated for 24 h. For the MTT test, MTT solution (5 mg ml^−1^) was added into each well and was incubated for another 3 h at 37 °C. The formazan product was dissolved in 100 μL per well DMSO after removing the media. The optical density (OD) was noted at 540 nm on the ELISA plate reader. The experiments were performed in triplicates for each concentration of formulation [17].

#### 2.9.2 Brine shrimp lethality assay

Now-a-days, to predict the cytotoxicity effect of bioactive compounds, the brine shrimp (sea monkey, artemia Salina or fairy shrimp) test (BST) is commonly used. It is the primary method used for screening the toxicity of plant extract, pesticides, dental materials, fungal toxins, and heavy metals. The brine shrimp lethality test was done by using the Meyer et al. method with some modifications [18]. Briefly, 1 mg per ml stock solution of the formulation was prepared by dissolving them in water. Different concentrations of (1 μg, 10 μg, 30 μg, 60 μg, 120 μg, 240 μg, 1000 μg, and 2500 μg) formulations were prepared from the stock solution and were put in their respective vials. Each tube contained 10 nauplii. The volume in each tube was adjusted to 5 ml by using artificial seawater (Himedia). Each concentration level was tested in triplicates. The negative control contained nauplii along with sterile Milli-Q and artificial seawater only. The tubes were incubated under a light source for 24 h. After 24 h, the dead nauplii in each tube were counted. Percentage motility (death) was calculated, and LC50 values were examined by using Prism software. An LC50 value higher than 1000 μg ml^−1^ was considered a non-toxic compound and was calculated from the graph [18].

### 2.10 Statistical analysis

All the experiments were performed in triplicates. The values are represented as mean ± SD (Standard Deviation). Statistical analysis of the obtained results was analyzed by using the software GraphPad Prism, and other significant values were calculated by one-way ANOVA; only values at p < 0.05 were considered significant.

## 3 Results

### 3.1 Anti-bacterial activity and MIC of formulation

An herbal formulation is a composition of more than one herbal extract, which is designed for the treatment of shigellosis. The anti-microbial activity of the designed formulation was examined against the *S. flexneri*, and a clear halo inhibitory zone of 23 mm was observed. According to the obtained result, the MIC value of the designed herbal formulations was found 2.4 mg ml^−1^ against the *S. flexneri*, and further experiments were performed at sub-lethal concentration.

### 3.2 Effect of formulation on the growth of *S. flexneri*

**Fig. 1:**
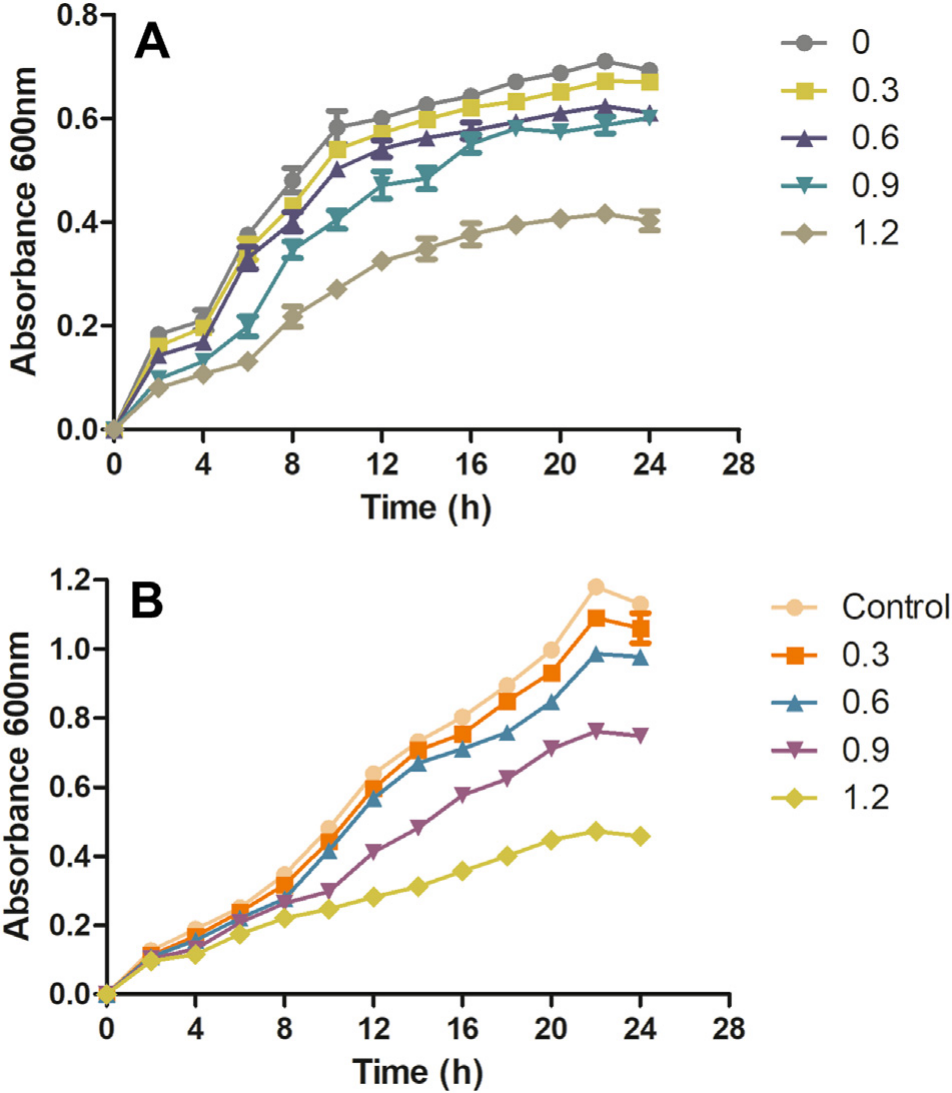
Growth curves of *S.flexneri* (1a) and *C. violaceum* CV026 (1b) at a sub-lethal concentration of formulation was plotted with respect to the control (no formulation). Error bars indicate the ± standard deviations of three measurements.

The effects of formulation (at sub-lethal concentration) on the growth curves of *S. flexneri* and *C. violaceum* CV026 were analyzed. The results showed that even though lower concentrations of formulation (0.3 and 0.6 mg ml^−1^) did not significantly affect the growth curves but, the concentrations of formulation higher than 0.6 mg ml^−1^ reduces the growth rate and will affect the other bacterial processes related to QS (Fig. 1).

### 3.3 Formulation inhibits the biofilm formation

**Fig. 2:**
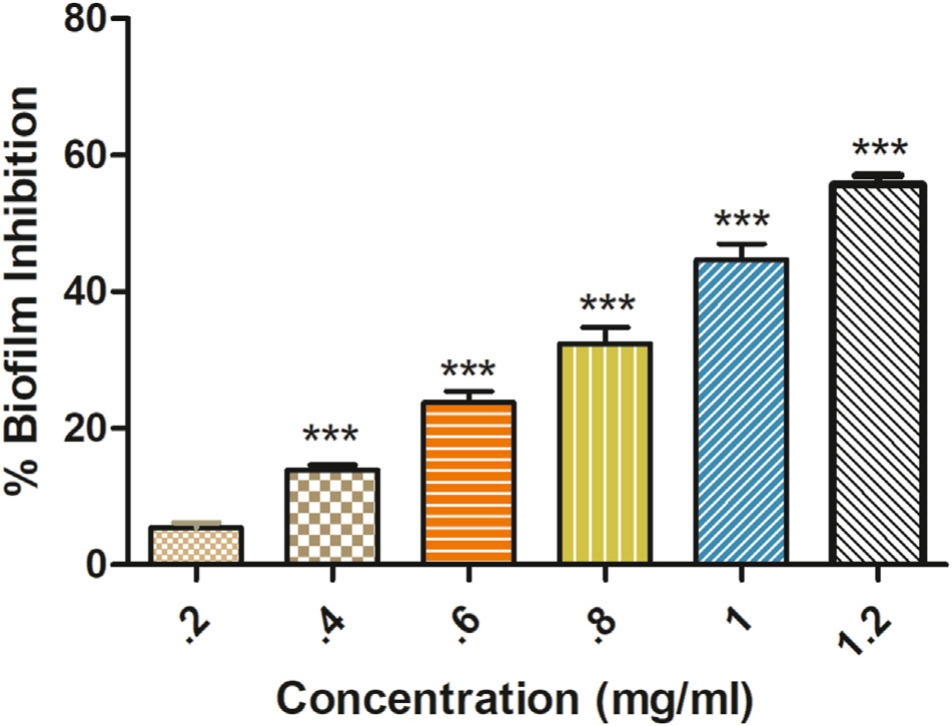
The graph is showing the % *Shigella* biofilm inhibition by designed formulation. Error bars indicate the ± standard deviations of three measurements. ∗∗∗ indicates the samples which were significant (one-way ANOVA *P < 0.001*).

Biofilm quantification was done to analyze the effect of formulation on biofilm formation. In the crystal violet binding assay, it was observed that the formulation inhibits the bacterial biofilm formation at 0.6 mg ml^−1^ concentration (23.78% inhibition) significantly when compared with the control (Fig. 2).

### 3.4 Quorum sensing inhibition

**Fig. 3:**
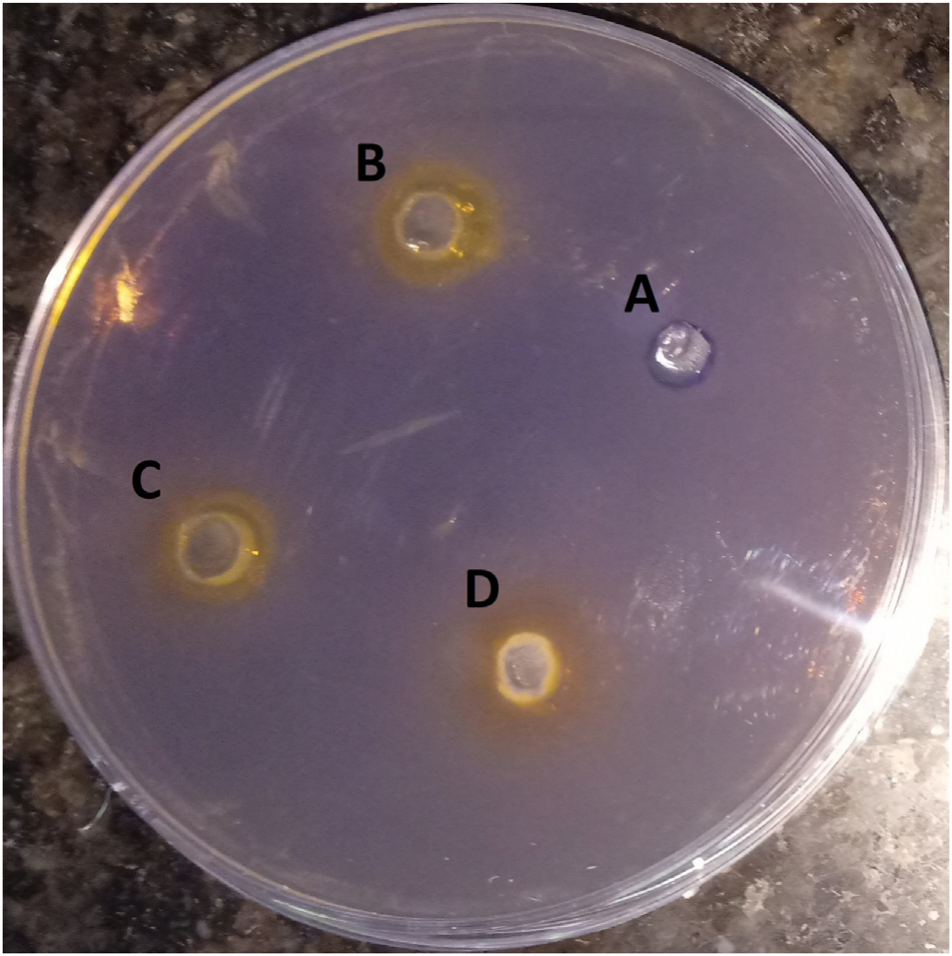
A plate is showing QS inhibition of *C*. *violaceum* CV026 by formulation, where A-control and B (0.2 mg/ml), C (0.3 mg/ml), and D (0.4 mg/ml) show the anti-QS activity of the formulation.

**Fig. 4:**
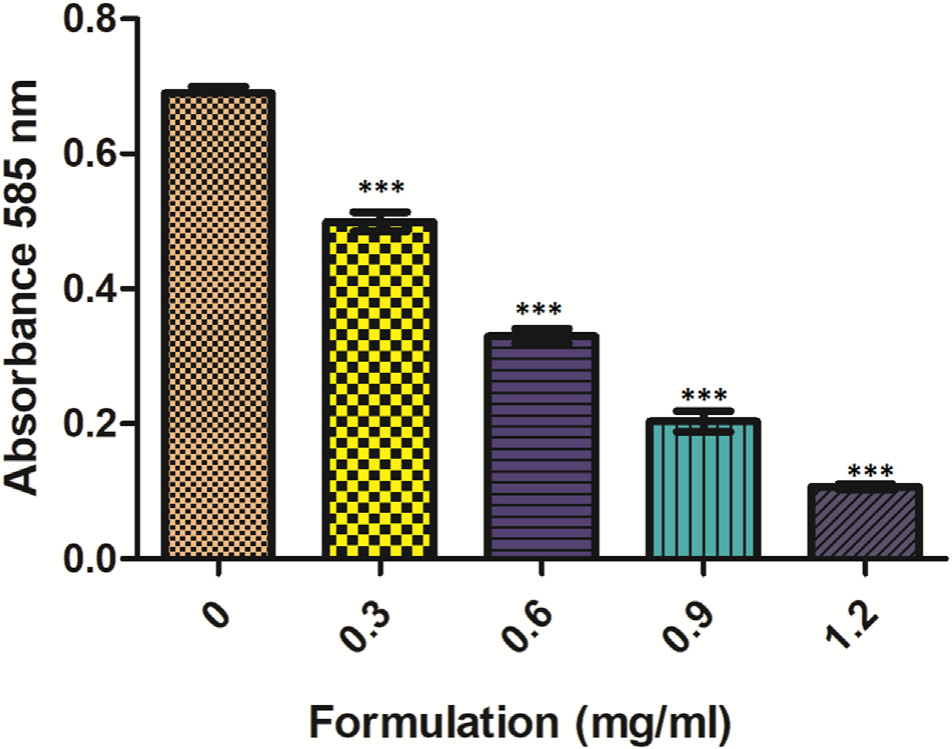
Inhibition of violacein production by formulation. Violacein production was noted spectrophotometrically at 585 nm. Values are presented as mean ± SD. ∗∗∗ indicates the samples which were significant (one-way ANOVA *P < 0.001*).

The designed formulation inhibitory activity against bacterial QS was analyzed by using violacein production (*C. violaceum* CV026). In the spread-plate method, a clear halo zone around the wells was observed, confirming the anti-QS activity of the designed formulation (Fig. 3). While in the quantitative inhibition assay, formulation shows concentration-dependent violacein inhibitory activity. Violacein production was found to be reduced by 27.68% at 0.6 mg ml^−1^ formulation concentration. With increasing concentration, the violacein production reduced to 84.55% at 1.2 mg ml^−1^ formulation concentration. This is maybe due to the combination of quorum sensing inhibition and reduced growth rate (Fig. 4).

### 3.5 Formulation cytotoxicity analysis

**Fig. 5:**
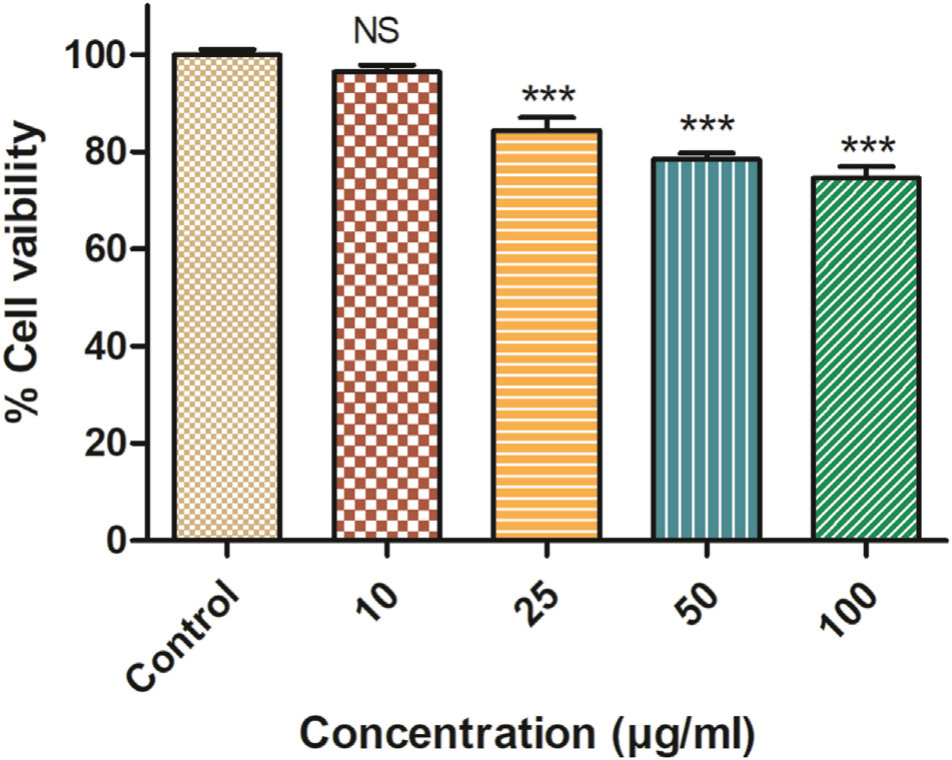
Cytotoxicity analysis of designed formulation by using SW480 cell line. All values are expressed as the mean ± SD of the three independent experiments. ∗∗∗ indicates the samples which were significant (one-way ANOVA *P < 0.001*).

Further, the cytotoxicity of the formulation was analyzed by carrying out MTT assay using cell line SW480 (Fig. 5), indicating concentration-dependent killing.

The brine shrimp test result is indicated in Table 1.

**Table 1:** The brine shrimp lethality assay of the formulation.

S.No	Concentration	No. of dead nauplii (initial average)	% Death/mortality (after 24 h)
			Formulations
1.	Control	00	0
2.	0.001 mg ml^−1^	00	0
3.	0.01 mg ml^−1^	00	0
4.	0.03 mg ml^−1^	00	10
5.	0.06 mg ml^−1^	00	13
6.	0.12 mg ml^−1^	00	20
7.	0.24 mg ml^−1^	00	33
8.	1 mg ml^−1^	00	57
9.	2.5 mg ml^−1^	00	100

According to Meyer [18], if the LC50 value is higher than 1000 μg ml^−1^, then the sample is considered as non-toxic and vice versa. The obtained results showed that the formulation designed was non-toxic to the nauplii, giving an LC50 value above 1000 μg ml^−1^ shown in Table 2. The tube containing only nauplii is taken as control.

**Table 2:** The obtain LC50 value of different formulations.

S.No	Formulation	Regression Equation	LC50 μg ml^−1^	Regression Coefficient (r2)
1.	F	Y = 37.84x + 10.38	>1 mg (1047 μg ml^−1^)	0.9340

## 4 Discussion

Shigellosis continues to cause havoc worldwide, with a high infectivity rate. It is responsible for bloody diarrhea and bacillary dysentery in developing countries, and among them, about 70% of deaths and approximately 60% of deaths in children under the age of five years. In developing countries, *S. flexneri* is mainly responsible for causing shigellosis [1,2]. The stomach cramps, fever, and blood in stool during shigellosis are due to the Shiga-toxin action [3]. From ancient times, herbal formulations have been used as an alternative therapy in different countries for the treatment of gastrointestinal disorders [19,20]. A formulation with components *C. sinensis* (leaf), *C. lemon* (fruit), *T. arjuna* (bark), *P. emblica* (fruit), and *T. chebula* (fruit) was designed for the shigellosis treatment. In this study, the anti-shigellosis activity was investigated on *S. flexneri* growth and its biofilm formation. The formulation was dissolved in an aqueous solvent. The major active compounds found in different extracts are tannin in *T. chebula*, polyphenols in *C. sinensis*, tannin in *P. emblica*, flavonoids in *C. lemon,* and flavonoids in *T. arjuna*. The *in* *vitro* results reveal that the formulation inhibits the *S. flexneri* growth with a MIC value of 2.4 mg ml^−1^. The clear halo inhibitory zone (23 mm) was observed, revealing the anti-bacterial activity of the formulation [16]. On analyzing the result of the individual component of the formulation and the designed formulation, an enhanced efficacy was observed in a designed formulation.

Further, sub-lethal concentrations of formulation were used because these concentrations do not affect bacterial growth. No significant difference was seen in the growth patterns between the control and formulation treated *S. flexneri* and *C. violaceum* CV026 at concentrations as low as 0.3 and 0.6 mg ml^−1^ in the growth curve analysis. The growth curve results reveal that the low formulation concentration (0.3 and 0.6 mg ml^−1^) inhibits QS, while the higher concentrations will affect both QS as well as the growth rate [11]. The lower concentration of 0.6 mg ml^−1^ formulation inhibits the bacteria biofilm formation (23.78% inhibition) significantly when compared with the control and reduces the violacein production (27.68%) compared with the control. This confirms the *in vitro* anti-quorum activity of the formulation (analyzed by using CV026). It also confirms the anti-biofilm activity of the formulation against the *S. flexneri* [11,15]. Similar data were also obtained by Noubissi et al. (with *Crinum jagus*), Wambe et al. (with *Cola anomala*), and by Limsuwan et al. (with Thai herbal formulation) [[21], [22], [23]]. The cytotoxicity analysis of the compounds by using a cell line is a widely accepted method. Cell line SW480 was used to determine the cytotoxicity of the designed formulations, and the results showed that the formulation is non-toxic or shows minor toxicity at a higher dose [17]. The brine shrimp test was commonly used for investigating the cytotoxicity of compounds. According to Meyer et al., if the LC50 value is higher than 1000 μg ml^−1^, the sample is considered non-toxic and vice versa [16]. The obtained result clearly shows that the formulation designed was non-toxic to the nauplii, giving an LC50 value above 1000 μg ml^−1^ [16].

## 5 Conclusion

The finding reveals that the formulation has anti-microbial, anti-biofilm, anti-QS activity, and was also found non-toxic. The evidence supports the anti-shigellosis effects of the designed formulation. However, in the future, *in* *vivo* and clinical investigation should be performed to support its efficacy further.

## Source(s) of funding

The author acknowledges the 10.13039/501100001411Indian Council of Medical Research (AMR/ADHOC/184/2019-ECD-II) support to execute the work.

## Conflict of interest

None.

## Author contributions

**Devendra Singh:** Conceptualization, Methodology, Writing- Original draft Preparation, Visualization, Investigation.

**Deepmala Sharma:** Analyzed the data. Vishnu Agarwal: Supervision.
