# Supplementary material for: Screening of anti-microbial, anti-biofilm activity, and cytotoxicity analysis of a designed polyherbal formulation against shigellosis
Source: J Ayurveda Integr Med. 2021 Nov 9;12(4):601–6. doi: 10.1016/j.jaim.2021.06.007 (PMC8642668; doi:10.1016/j.jaim.2021.06.007)
Supplement: Multimedia component 1 [file mmc1.docx]

**Table 1:** MICs values of individual extracts

| S.No | Aqueous Extracts | MIC value against *S.flexneri* |
| --- | --- | --- |
| 1 | *Camellia sinensis* | > 4.2 mg.ml^-1^ |
| 2 | *Phyllanthus emblica* | 3.2 mg.ml^-1^ |
| 3 | *Citrus lemon* | 3.6 mg.ml^-1^ |
| 4 | *Terminalia arjuna* | > 4.2 mg.ml^-1^ |
| 5 | *Terminalia chebula* | > 4.2 mg.ml^-1^ |
